# Pathological Presence of Free Air in the Thorax: Pneumothorax and Pneumomediastinum as a Complication of COVID-19

**DOI:** 10.7759/cureus.40996

**Published:** 2023-06-26

**Authors:** Alexandra M Cristea, Dragos C Zaharia, Stefan Dumitrache-Rujinski, Alexandra Tintea, Miron A Bogdan

**Affiliations:** 1 Department of Pneumology, Carol Davila University of Medicine and Pharmacy, Bucharest, ROU; 2 Department of Pneumology, Marius Nasta Institute of Pneumology, Bucharest, ROU; 3 Department of Public Health, Carol Davila University of Medicine and Pharmacy, Bucharest, ROU

**Keywords:** pandemic, subcutaneous emphysema, pneumothorax, pneumomediastinum, covid-19

## Abstract

Introduction: The abnormal presence of free air in the thorax, pneumothorax, and pneumomediastinum are complications for critically ill patients suffering from coronavirus disease 2019 (COVID-19). The development of these events may lead to a poor prognosis and make the management of this category of patients more difficult.

Study design: We performed an observational retrospective study, including patients with SARS-CoV-2 infection and pneumonia who were hospitalized, to analyze the cases that developed pneumothorax or pneumomediastinum as a complication.

Results: A total of 28 cases (1.51%) from 1844 patients with SARS-CoV-2 pneumonia developed pneumothorax or pneumomediastinum during hospitalization. Of them, 21 (75%) needed intensive care unit admission and ventilation, and 10 (35.71) were cured.

Conclusion: The male gender is more probable to be involved in the development of pneumothorax or pneumomediastinum in patients with SARS-CoV-2 pneumonia. The incidence of these events is low, and conservative treatment could provide a better outcome.

## Introduction

Pneumothorax and pneumomediastinum are rare complications occurring during hospitalization in patients with coronavirus disease 2019 (COVID-19), with a low incidence. A study including 4500 patients found that the incidence was around 1%, with 39 developing pneumothorax and seven developing pneumomediastinum [1]. There are two mechanisms that explain this pathological condition: one is the Macklin phenomenon, i.e., the destruction of the alveolar walls as a consequence of the cytokine effect, leading to the spontaneous development of the pneumothorax or pneumomediastinum, and the other mechanism is secondary to barotrauma and volume trauma, especially in mechanically ventilated patients [2].

Cough is one of the most common symptoms in patients with COVID-19, along with fever, fatigue, and dyspnea [3], and it may be present from the early stages of mild disease, to moderate cases of pneumonia and in severe and critical cases that developed acute respiratory distress syndrome (ARDS), an important factor of severity for the poor prognosis and admission to intensive care unit (ICU) [4]. Intense cough with the presence of inflammation leads to direct alveolar injury with the rupture of the alveoli, leading to the spread of the air within the mediastinum [5].

Despite the low incidence, these complications are associated with a poor prognosis and higher mortality and risk for mechanical ventilation in patients with COVID-19 and pneumonia [6].

The aim of our study was to determine the characteristics, such as previous pulmonary disease, gender, and symptoms if they were mechanically ventilated, of patients with SARS-CoV-2 pneumonia who developed complications such as pneumothorax and pneumomediastinum during hospitalization.

## Materials and methods

This is a retrospective observational study described before in an article where we analyzed complications in hospitalized patients with SARS-CoV-2 pneumonia [7]. This study was approved by the Marius Nasta Institute of Pneumology Ethics Committee and all the patients signed the informed consent during hospital admission.

The diagnosis of pneumothorax or pneumomediastinum was confirmed by chest radiography or chest computed tomography (CT), or by clinical examinations associated with symptoms, with an increase in the severity of symptoms, a drop in oxygen saturation, and the clinical evidence of subcutaneous emphysema.

The inclusion criteria were as follows: SARS-CoV-2 infection confirmed by reverse transcription-polymerase chain reaction, rapid antigen, or serum antibodies, and lesions suggesting pneumonia, pneumothorax, and pneumomediastinum on imaging (chest radiography or CT).

All the data were analyzed in Microsoft Excel (Microsoft Corporation, Redmond, WA) and we obtained quantitative, percentage, and numerical variables from the discharge documents of the patients, and chest CT scans from the hospital's database.

## Results

Out of 1844 patients admitted with SARS-CoV-2 pneumonia, 28 (1.51%) developed pneumothorax or pneumomediastinum during hospitalization. The characteristics of these patients are shown in Table 1. The mean age was 59.71 years (with a range of 46 to 78 years) and 78.57% were males.

**Table 1:** Characteristics of the patients who developed pneumothorax or pneumomediastinum.

Characteristics		Number	Percentage (%)
Gender	Male	22	78.57
	Female	6	21.42
Type of complication	Pneumothorax	12, with 3 hydropneumothorax	42.85
	Pneumomediastinum	16	57.14
Previous pulmonary disease	Yes	6	21.42
	No	22	78.57
Symptoms at admission	Cough	16	57.14
	Dyspnea	24	85.71
	Fever	10	17.24
Intensive care unit admission	Yes	21	75
	No	7	25
Associated complications	Subcutaneous emphysema	8	28.57
	Pulmonary thromboembolism	5	17.85
	Others (pneumoperitoneum, bacterial infection)	7	25
Evolution	Cured	10	35.71
	Died	18	64.28

Out of 28 cases, 16 developed pneumomediastinum and three cases developed hydropneumothorax, of which two cases already had a pleural effusion at the time of hospital admission. Six patients had the following pulmonary diseases previously: pulmonary cancer associated with pleural effusion, pleural effusion drained, pulmonary thromboembolism, interstitial lung disease, asthma, and bronchiectasis.

The most frequent symptom at admission was dyspnea with 85.71% of the total number of cases, followed by cough (57.14%) and fever (17.24%). The intensive care unit admission was 75% and 64.28% of cases had a poor prognosis.

Regarding the management of these patients, pneumothorax was treated surgically, with the insertion of a drainage tube in the pleural cavity. Pneumomediastinum was treated conservatively. In all the cases, the major action taken was to lower as much as possible the respiratory volume, oxygen flow, and ventilation pressure, to ensure an optimal oxygen saturation over 90%. Of the 21 patients admitted to the ICU, four were cured, and all the patients from the ICU were ventilated invasively or noninvasively.

**Figure 1:**
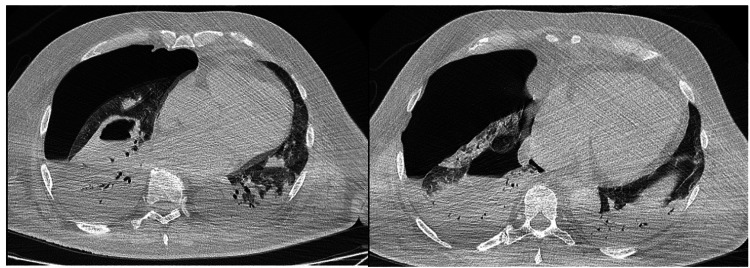
Chest computed tomography scan of a 63-year-old male with severe SARS-CoV-2 pneumonia and spontaneous right hydropneumothorax, with no previous chronic pulmonary disease.

Figure 1 shows the chest CT of a patient with SARS-CoV-2 pneumonia and right spontaneous hydropneumothorax. The patient was 63 years old with no previous pulmonary disease and with severe lesions of pneumonia.

**Figure 2:**
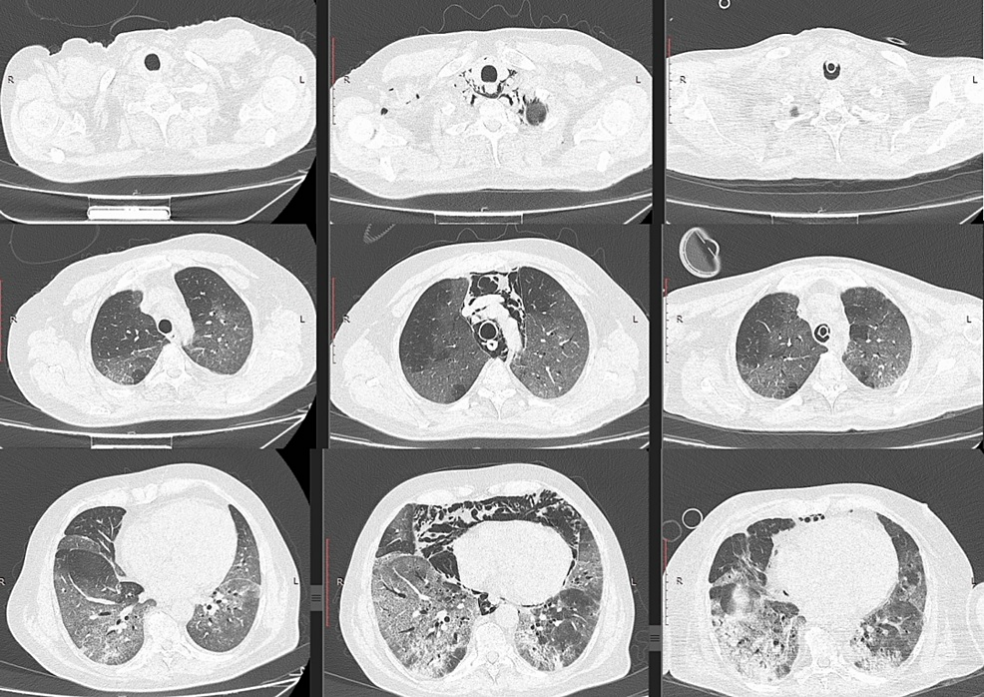
Series of images of computed tomography scan of a 51-year-old male with severe pneumonia done on days three, 10, and 15 after admission. The presence of spontaneous pneumomediastinum and subcutaneous emphysema on day 10 is notable, with the evident regression of the lesions on day 15, even when intubation and mechanical ventilation were needed.

Figure 2 shows a series of chest CT images of a patient with SARS-CoV-2 pneumonia and pneumomediastinum associated with subcutaneous emphysema; the investigations were performed days apart. It is notable that the air from the mediastinum disappeared in five days, even when the patient was intubated and mechanically ventilated.

## Discussion

The abnormal presence of free air inside the thorax can be localized inside the mediastinum structures, pneumomediastinum (PM), or between the pleural and visceral pleura, producing a pneumothorax (PTX). In many cases, the development of such a condition may be spontaneous but many factors can be incriminated in the pathology of a secondary PM, including medical procedures (for example, intubation or central vascular access procedures), trauma (penetration of chest), or non-traumatic (asthma, chronic obstructive pulmonary disease, bronchiectasis, malignancy, or infection) [8]. One study, including 112 patients with ARDS by infection, found that 13 cases developed spontaneous pneumomediastinum (11.6%) and concluded that this is a frequent complication in cases of ARDS [9]. Also, there are some cases of secondary pneumomediastinum reported in cases of viral pneumonia produced by H1N1 influenza and severe acute respiratory syndrome coronavirus 1 (SARS-CoV-1) [10].

The incidence of PTX or PM in COVID-19 patients is low. We found 28 cases (1.51%) out of 1844 hospitalized patients with SARS-CoV-2 pneumonia. The incidence rate of subcutaneous emphysema and spontaneous pneumomediastinum is 3.0 and 1.2 per 100,000 non-intubated COVID-19 patients, respectively [11]. A study including 58,484 patients hospitalized with COVID-19 found an incidence of 0.64% and concluded that this is a marker of severe pneumonitis with no association with mechanical ventilation [12]. Another study including 427 patients admitted to the ICU found an incidence of 5.6% [13]. The incidence may increase up to 15% with mechanical ventilation as a consequence of barotrauma [14], but there are contradictory studies supporting this theory. Figure 2 shows a series of CT images of a 51-year-old patient with severe COVID-19 and pneumonia, performed three, 10, and 15 days after admission. On day 10 of hospitalization, the CT scan performed shows the presence of air in the mediastinum and subcutaneous emphysema in the cervical region, with modifications that regressed on day 15, even when the patient was intubated and mechanically ventilated.

In our study, almost 80% of the cases were male, and the median age was around 59 years. These findings are consistent with the ones from a study conducted on 10,605 patients with COVID-19 hospitalized during the first and second wave of the pandemic, of which 66 developed PM, 83% were males, and the mean age was 52 years [15]. The majority of patients developing PM are male in 75% of cases, and it seems that in 70% of the patients, subcutaneous emphysema is associated [16].

Although chronic pulmonary pathology is incriminated in the development of PTX or PM, in the case of patients with COVID-19, this is not considered a risk factor. A study conducted on patients admitted to the ICU with a critical stage of the disease showed that only three out of 13 patients suffered from pre-existing lung disease [17]. Those findings are similar to our study. We found that 78.57% of the cases did not have a previous chronic pulmonary disease. However, it seems that the development of pneumonia during SARS-CoV-2 infection may be a risk factor for the development of spontaneous pneumomediastinum [18].

Cough is a symptom present in patients with SARS-CoV-2 pneumonia, and the increased pressure in the chest that it determines may explain the development of PTX or PM [19]. But cough is also a symptom present in ARDS along with severe dyspnea [4], with a higher incidence of developing those types of complications [20]. Subcutaneous emphysema is associated with pneumomediastinum in 28.57% of cases. There are not much data in the literature about this complication, but the association of both is frequent. A study shows that out of 10 patients admitted to the ICU with severe COVID-19, all of them also developed subcutaneous emphysema during hospitalization [21]. Case reports from the literature are showing the association with other complications, such as pneumoperitoneum [22], venous thromboembolism [23], or bilateral pulmonary embolism [24].

A limitation of the study is represented by the fact that the cases included are patients with SARS-CoV-2 pneumonia hospitalized during the period consistent with the first three waves of the pandemic, and maybe it would be more relevant to include patients infected with Delta and Omicron variants of the viruses. Another limitation of the study is represented by the fact that we were not able to identify the moment when this complication occurred.

## Conclusions

Male gender may be a risk factor for the development of pneumothorax or pneumomediastinum in patients with COVID-19 and pneumonia. Even if mechanical ventilation (invasive or noninvasive) is reported as a risk factor for developing pneumomediastinum, in some cases with the correct management and low volume and pressure of the oxygen, it is possible to be ameliorated or even cured within days. Dyspnea is the major symptom associated with these patients, followed by cough and fever. Previous pulmonary disease is not a condition for the development of these complications.

Even if the incidence of pneumothorax and pneumomediastinum is low in patients with COVID-19, it is important to be aware of it, because in most cases, conservative measures are the only action needed for a better outcome.
